# Quantifying the Error Associated with Alternative GIS-based Techniques to Measure Access to Health Care Services

**DOI:** 10.3934/publichealth.2015.4.746

**Published:** 2015-11-18

**Authors:** Amy Mizen, Richard Fry, Daniel Grinnell, Sarah E. Rodgers

**Affiliations:** 1 The Centre for the Development and Evaluation of Complex Interventions for Public Health Improvement (DECIPHer), College of Medicine, Swansea University Medical School, Swansea, UK SA2 8PP; 2 Farr Institute, College of Medicine, Swansea University Medical School, Swansea, UK SA2 8PP; 3 Universities' Police Science Institute, Cardiff University School of Social Sciences, 1-3 Museum Place, Cardiff, CF10 3BD

**Keywords:** accessibility, GIS, public health, aggregation error, network distance, Euclidean distance, health inequalities

## Abstract

The aim of this study was to quantify the error associated with different accessibility methods commonly used by public health researchers. Network distances were calculated from each household to the nearest GP our study area in the UK. Household level network distances were assigned as the gold standard and compared to alternate widely used accessibility methods. Four spatial aggregation units, two centroid types and two distance calculation methods represent commonly used accessibility calculation methods. Spearman's rank coefficients were calculated to show the extent which distance measurements were correlated with the gold standard. We assessed the proportion of households that were incorrectly assigned to GP for each method. The distance method, level of spatial aggregation and centroid type were compared between urban and rural regions. Urban distances were less varied from the gold standard, with smaller errors, compared to rural regions. For urban regions, Euclidean distances are significantly related to network distances. Network distances assigned a larger proportion of households to the correct GP compared to Euclidean distances, for both urban and rural morphologies. Our results, stratified by urban and rural populations, explain why contradicting results have been reported in the literature. The results we present are intended to be used aide-memoire by public health researchers using geographical aggregated data in accessibility research.

## 1. Introduction

Providing equal access to health care is an important priority in international public health policy [1]–[7]. This is because equitable access to healthcare is strongly linked with reducing ill health and suffering [8]. There are several components to measuring accessibility but the geographical aspect of accessibility describes how easily a population can travel to health services. This measure is based on: 1) the distance people live from health services, 2) how good public transport links are to the health services and 3) how long it takes to travel to such services [9]. Equal geographical access to healthcare facilities is, however, unrealistic for public health planners and policy makers to attain [10]. Rather, health services are concentrated in more densely populated areas so to serve an optimum catchment of the population. Therefore, urban populations tend to have shorter distances to travel to health services compared to rural populations [11]. There is a growing need to understand the relationship between accessibility and health in order to lessen provision inequalities [12]. The extent to which people can access services needs to be accurately assessed and effectively communicated to planners and public health practitioners so that successful policy and infrastructure planning can be implemented.

Geographical Information Systems (GIS) can be used to model geographical accessibility to health services [10],[11],[13]–[16]. Common techniques used to calculate accessibility in public health research are Euclidean (straight line) and network distance measurements. More recently, sophisticated representations of accessibility modelling such as gravity models, kernel density models and 2-step floating catchment area models [17]–[20] have been published in the literature. However, among public health practitioners, Euclidean and network distances methods remain popular choices for modelling spatial accessibility to services [21]. It has been suggested that for some populations and geographies, the more basic Euclidian distance measure does not provide a sufficiently representative distance estimate [22]. Alternatively, the generation of network distances may be unnecessarily complex depending on the study context [23]. The aim of this paper is to quantify the error associated with Euclidean and network distance accessibility methods so that public health practitioners can quote quantified errors when they are undertaking research and understand the limitations of research methods.

In addition to distance type, origin and destination data types also influence the accuracy of the accessibility assessment. Ideally accessibility modelling would use address level data as an origin in origin-destination calculations [24]–[26]. However, most accessibility studies use spatially aggregate origin data because: 1) often they are the only available data; 2) as a way of protecting the privacy by collating individuals into non-identifiable spatial units; 3) aggregation reduces computational and storage requirements [27]. Aggregation units are typically defined by the number of people they contain which introduces ecological fallacy, whereby an inference about an individual is made based on the population to which that individual belongs. Larger spatial units represent larger populations and smooth local variation, often leading to erroneous results and misleading conclusions [28]. When only aggregate data are available, it is important that researchers are aware that error is introduced because of the introduction of ecological fallacy into statistical models, producing biased results [29]. The extent of aggregation error should be better documented [9],[30] so that the magnitude of error can be recorded and included in the analysis and interpretation of results.

In this study we have examined the potential access to General Practitioner (GP) surgery (Primary Care Physician) locations. In the UK there are no fees incurred per visit to the GP under the National Health Service (NHS), which is available to all, and an individual typically registers with a GP surgery near their home. We have used widely applied distance measures at four levels of aggregation, compared the different methodological approaches and quantified the error associated with each method. We discuss the implications of using inappropriate accessibility estimates, before recommending which methods should be used in different study contexts. We highlight the importance of assigning people to their correct facility, and the implications of assigning people to the wrong facility. This study includes a range of population geographies, several measurement techniques and rural and urban comparisons for a city with a different urban form to those found in North America.

## 2. Methods

### 2.1. Study Area

This study was set in the Swansea administrative area in the United Kingdom. Swansea is the second largest city in Wales, UK with a population of 240,300 [31] distributed amongst 109,640 households. The population is distributed across a variety of urban and rural landscapes with a population density ranging from 30 people per km^2^ to 6810 people per km^2^ [32]. The variability of Swansea's population distribution makes it representative of a typical UK population.

### 2.2. Data

**Figure 1.**
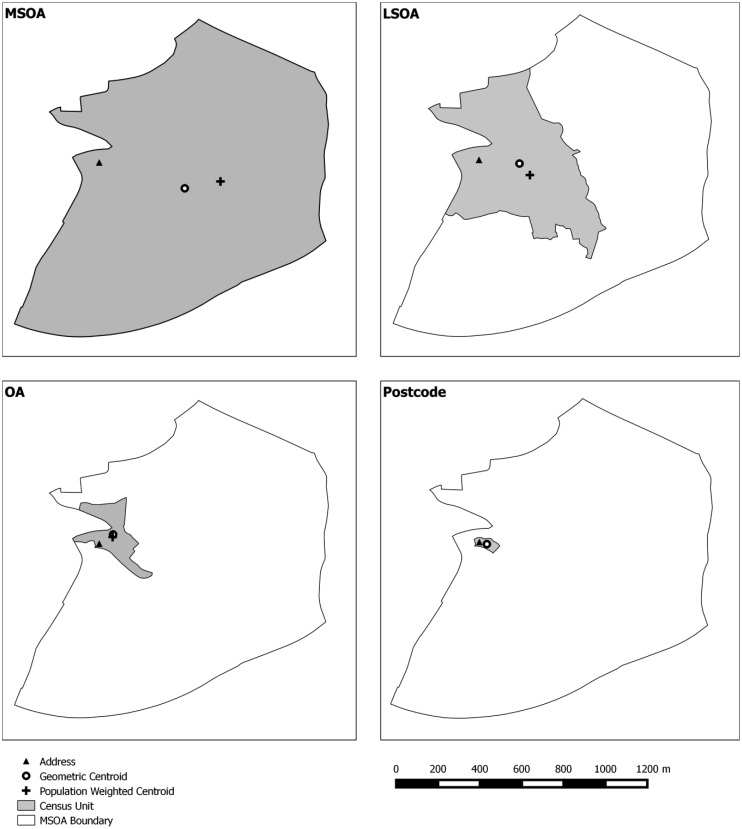
Census unit boundaries.

The 47 GP surgery locations in the Swansea administrative area were identified using the Ordnance Survey Points of Interest dataset and confirmed using the list on the NHS Wales Informatics Service Website [33],[34]. Residential address locations (n = 109,640) within the Swansea administrative area were extracted from AddressBase Premium [35]. Four commonly used spatially aggregated units of population were used to generate comparator data namely: Unit Postcode (the base unit of postal geographies in the UK), Output Area (OA), Lower Super Output Area (LSOA) and Middle Super Output Area (MSOA). Unit Postcode data from Code Point were supplied by the Ordnance Survey [36] and provided boundary polygons for each unit postcode. The OA, LSOA and MSOA aggregation units are from the 2011 UK Census of Population, Office for National Statistics [36]. Spatial units are designed to meet specific homogeneity criteria so that they are comparable by population size [37]. The different aggregation units used in this study are listed in Table 1, together with international equivalents, and their relative spatial coverage displayed in Figure 1.

Each LSOA was classified as rural or urban based on the rurality index generated by the Office of National Statistics [37]. Areas with less than 10,000 people were classified as rural and those with more than 10,000 people classified as urban. The road and footpath network was provided by the OS MasterMap Integrated Transport Network (ITN) Layer [38].

**Table 1.** Spatial aggregation units. Example of comparable international spatial units and the average population contained within

Spatial Unit	Average Population	Comparable International Units
**Unit Postcode**	50	Japan: *Prefecture*
** OA**	100	Australia: *Meshblock*
** LSOA**	1500	Japan: *Municipality*; USA: *Block Group*
** MSOA**	7500	USA: *ZIP Codes*; Australia: *SA2s*

### 2.3. GIS Methods

Distance measures were created at address level and the specified aggregation units using two GIS methodologies – network distances and Euclidean (straight line) distances. The network distance from each address and aggregation unit to the nearest GP surgery was measured in a GIS using the network route to create Origin-Destination (OD) matrices. For Euclidean distances, the ‘Near’ tool was used (ArcGIS™ 10.1).

Address level network distance was defined as the gold standard as it was most likely (methodologically) to represent the true distance between a residence and a GP surgery. For each unit of aggregation, population weighted and geometric centroids were used as the origin of the journey for the population represented within that unit. Population weighted centroids for OA, LSOA and MSOA were obtained from ONS [39]. Both centroid types were used in the analysis to assess the impact of the commonly used population weighted centroid on distance measures.

### 2.4. Statistical Methods

Due to the non-normal distribution of the data, a Spearman's Rank coefficient was performed using the raw distance data. This method was used to identify correlations between the different distance measures (spatial unit and different centroids) and the gold standard address-based network distance estimates. The address-based network distance estimates were used as a baseline against which all other distance and aggregation unit measurement methods were compared. The median distance refers to the median of distance measures from the centroid of a spatial unit to its nearest GP in the study area, and have been described as a distance error for the purpose of this study. Further to this, the proportion of homes that were assigned to an incorrect GP as the nearest GP was recorded. This was so that the impact of methodology and areal unit size on an individual's GP assignment could be assessed.

## 3. Results

The distance calculation method, level of spatial aggregation and centroid type are reviewed with comparisons made between each distance calculated and stratified against the rurality of the areal unit. Distance estimates and associated statistics are summarised for each distance method, and all spatial aggregation units and centroid types (Table 2). Error was reported as the difference between the gold standard distance and the modelled distances.

### 3.1. Distance measurement methods

**Figure 2.**
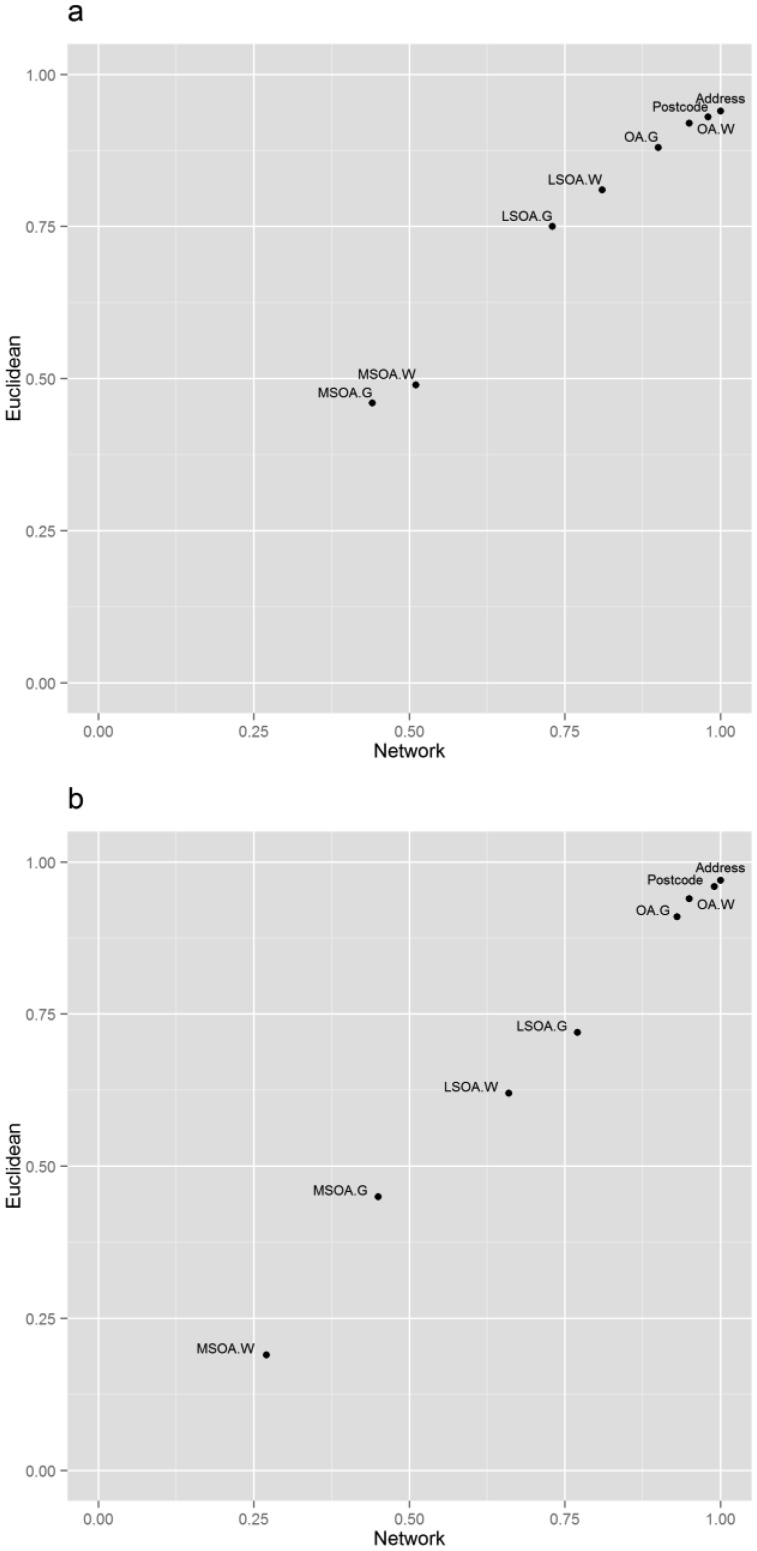
Relationship between Euclidean and network distance measures. (G, geometric; W, population weighted): (a) Urban morphologies (b) Rural morphologies

The network distance methodology produced a wider range of distances than Euclidean distances. This is demonstrated by a larger interquartile range (IQR, Table 2). Despite the larger IQR, network distances produced smaller error margins relative to the gold standard. In contrast, the Euclidean distance measures result in a smaller IQR, but larger error margins than network distances. The correlation between Euclidean and network distances were assessed using Spearman's rank (Figure 2). Each distance measure was compared to the gold standard measure. The plots of the ρ coefficients reveal that Euclidean and network distances have a positive linear relationship at each level of spatial aggregation. All distance measures were found to be significantly related to the gold standard (*p* < 0.01). However, the Spearman's ρ coefficient values indicates the strength of the relationship ranges from weak (0.19 for the largest areas (MSOA)) to strong (0.99 for the smallest areas (Unit Postcodes)). The ρ coefficient values have a greater range in rural areas than urban areas (Figure 2(b)).

**Figure 3.**
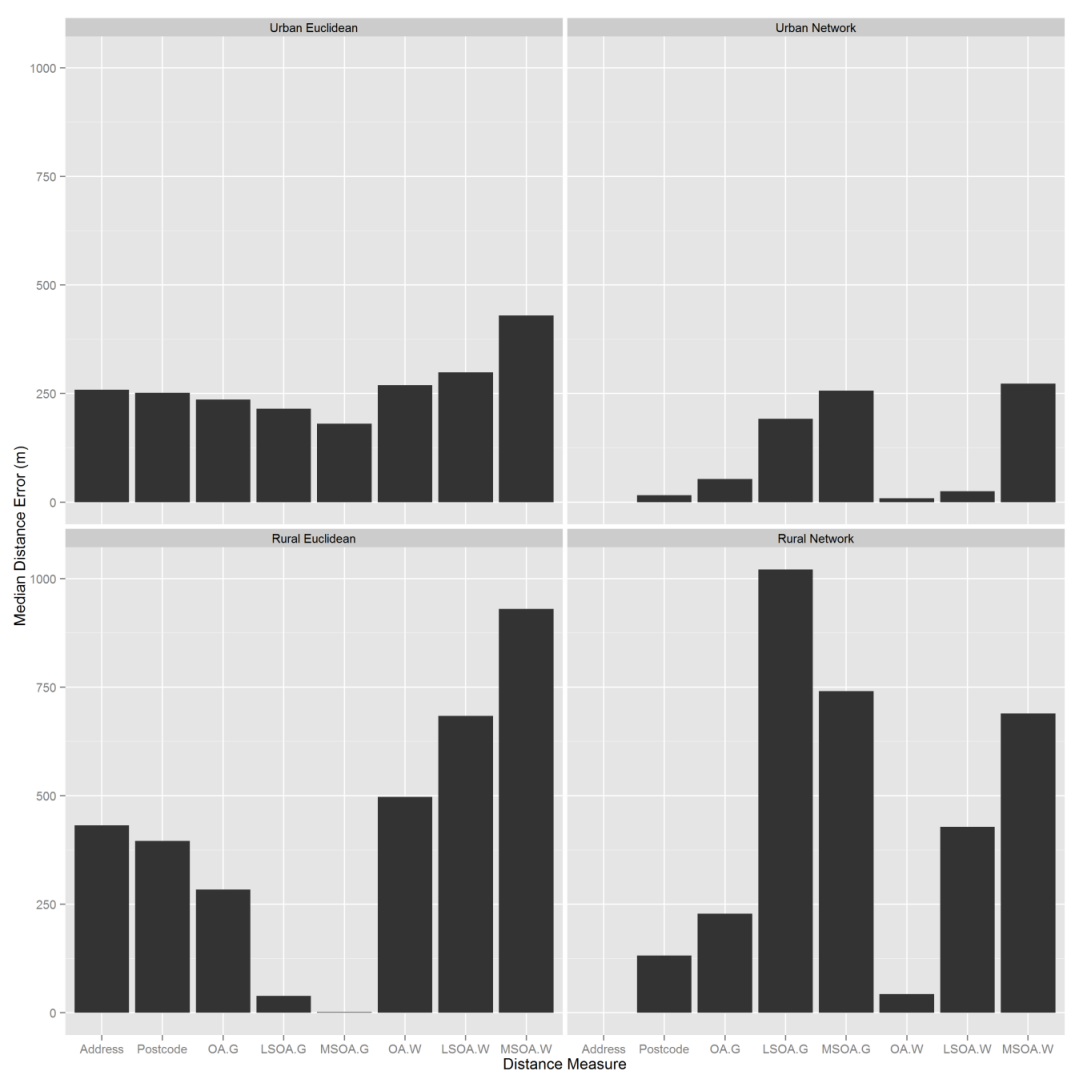
Median distance errors

For urban areas, Euclidean distance errors were greater than network distance errors. However, in rural regions, Euclidean distances have far smaller distance errors when using geometric centroids (Figure 3). Overall, Euclidean and network distance errors are smaller for urban regions compared to rural regions.

### 3.2. Distance measurement errors resulting from spatial aggregation

Urban areas recorded smaller distance errors for all levels of spatial aggregation than rural areas for every distance type (Figure 3). The maximum error was for LSOA Euclidean distances in urban areas (485m) and LSOA Network distances in rural areas (1021m). As the level of spatial aggregation increased, the distance errors for both network and Euclidean methods increased compared to the gold standard. For data aggregated at the MSOA level, although they are not the largest distance errors, there is an overall correlation of less than 0.5 with the gold standard, indicating that neither distance method is an acceptable solution for data aggregated at the MSOA level.

### 3.3. Distance measurement errors resulting from centroid type

The use of population weighted centroids with the network distance method in urban areas produced smaller distance errors than geometric centroids. For urban Euclidean distances, distance errors did not vary much between centroid type (Figure 3). In rural regions, at LSOA and MSOA level, geometric centroids produced the greater distance errors when combined with network distances. In contrast, for Euclidean distances, population weighted network distances produced greater errors than Euclidean geometric distances.

**Table 2.** Median, Interquartile Range (IQR) and Maximum distance estimates (metres).

		*Address*	*Unit Postcode*	*OA.G*	*LSOA.G*	*MSOA.G*	*OA.W*	*LSOA.W*	*MSOA.W*
	***Euclidean***								
***Urban***	*Median*	590	597	613	634	668	580	550	419
	*IQR*	623	623	649	737	778	623	608	340
	*Max.*	3,134	3,100	3,039	2,964	4,704	2,896	2,875	2,972
	***Network***								
	*Median*	849	865	902	1,041	1,106	840	824	576
	*IQR*	829	836	895	922	1,170	818	790	578
	*Max.*	4,552	5,048	4,202	3,859	6,815	4,544	3,606	3,404
***Rural***	***Euclidean***								
	*Median*	1,377	1,413	1,525	1,770	1,811	1,312	1,125	879
	*IQR*	1,767	1,767	1,892	2,026	891	1,847	1,962	2,365
	*Max.*	7,255	8,329	6,060	5,532	4,704	6,961	4,683	3,384
	***Network***								
	*Median*	1,809	1,941	2,037	2,830	2,550	1,766	1,381	1,120
	*IQR*	2,236	2,293	2,499	2,410	960	2,321	2,199	2,405
	*Max.*	11,410	12,270	9,854	10,220	6,815	9,107	8,018	4,002

Geometric centroids (G) and population weighted (W) centroids are compared for all distance methods (Euclidean and network) by morphology (urban or rural).

**Figure 4.**
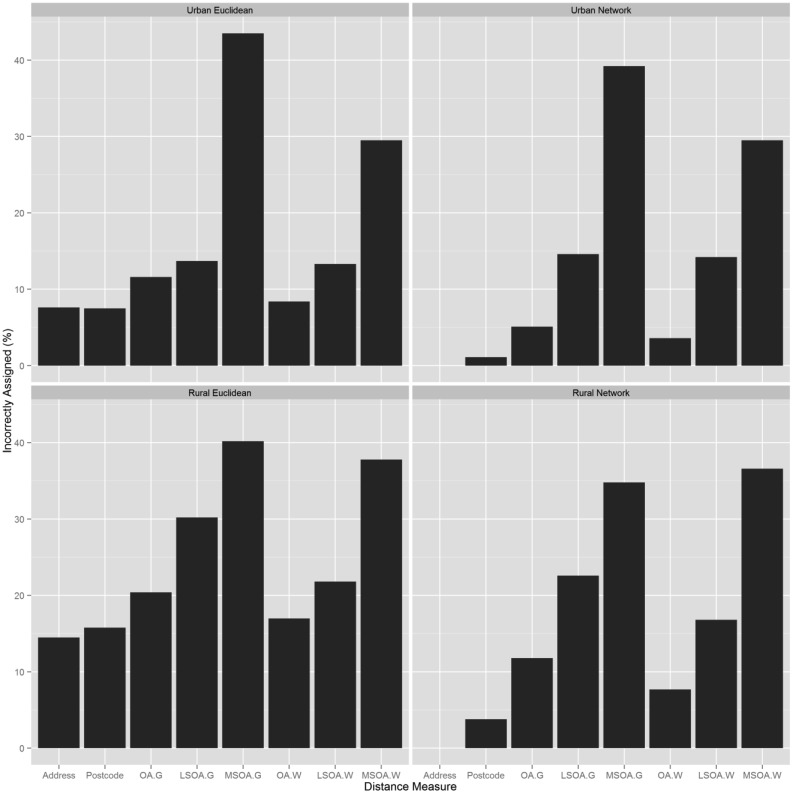
Nearest facility assignment errors

**Table 3.** Nearest facility assignment error.

Urban									
			Unit Postcode	OA.G	LSOA.G	MSOA.G	OA.W	LSOA.W	MSOA.W
Network	n	0	3,650	11,427	21,926	33,755	7,327	16,329	35,534
	**%**	**0**	**3.8**	**11.8**	**22.6**	**34.8**	**7.7**	**16.8**	**36.6**
Euclidean	n	14,047	15,368	19,747	29,280	39,021	16,456	21,110	36,654
	**%**	**14.5**	**15.8**	**20.4**	**30.2**	**40.2**	**17.0**	**21.8**	**37.8**

Rural									
			Unit Postcode	OA.G	LSOA.G	MSOA.G	OA.W	LSOA.W	MSOA.W
Network	n	0	138	642	1846	4946	459	1793	3713
	**%**	**0**	**1.1**	**5.1**	**14.6**	**39.2**	**3.6**	**14.2**	**29.5**
Euclidean	n	960	948	1457	1731	5489	1064	1678	3713
	**%**	**7.6**	**7.5**	**11.6**	**13.7**	**43.5**	**8.4**	**13.3**	**29.5**

### 3.4. Nearest Facility Identification

Address-based network distances were assumed to have resulted in 100% of people assigned correctly to their nearest GP. Relative to this, the number of GPs incorrectly assigned to households increased as the spatial unit size increased (Table 3, Figure 4).

At every spatial unit, network distances correctly assigned more households than Euclidean distances. The largest error occurred when a Euclidean distance method was used with a geometric centroid for MSOA's resulting in 44% of households incorrectly assigned to the correct GP. Using a population weighted centroid decreased the number of people incorrectly assigned to the nearest GP by more than 10% when using OA or LSOA data. Residents were more likely to be assigned to an incorrect GP if they lived in a rural area. The Spearman's rank ρ value for the address-based network distance method and urban OA for network and Euclidean distances, was 0.90 and 0.91 respectively. However, in practical terms 11% or 11,427 people were assigned to the wrong GP using the network method, rising to 20% or 19,747 people using the Euclidean distance method. At every level of aggregation, the more complex the distance method, the lower the rate of incorrect assignment. Rural Euclidean distances had higher rates of incorrect assignment than network distances. In LSOAs where there were no GP surgeries, over 75% of residents were incorrectly assigned with Euclidean distances, compared to 30–50% for network distances.

## 4. Discussion

This study has demonstrated that measuring access to services, such as GP's, can be complex and result in a wide range of accessibility measures, depending on the methodology and data used.

Previous research that investigated distances to hospitals in the USA found little difference between Euclidean and network distance methods [23]. However, we recommend that network measures should be used in favour of Euclidean measures whenever possible. In large urban areas it could be argued that Euclidean distances are an adequate proxy for the distance travelled. Urban areas have greater concentrations of people living in close proximity to each other and there is greater connectivity in road and footpath networks. Increased connectivity allows the population to move more directly around the area in which they live, i.e. there is more opportunity to travel the “Euclidean route”. The increased street connectivity combined with smaller geographical areas covered by the aggregation unit (compared to rural areas) results in the Euclidean distance acting as a reasonable proxy for network distances. Euclidean measures should be used with caution as they do not take into account topographic considerations and can result in environmental exposures being lost or masked. For example, rivers, railway lines and motorways are barriers which can have a great impact on an individual's ability to access a service. Such barriers can be accounted for with network distances. Using network distances over Euclidean distances will be particularly relevant where road networks have evolved differently to a planned grid based system like those in North America and Australia. Network distances and routes provide greater detail about the local environment that people experience when travelling to reach their destination compared to Euclidean distances. Future research will be able to provide important information about exposures within the environment, which could be used to contextualise data and better understand social behaviours. These are important considerations for progressing towards developing accessibility models that model a realistic journey that is taken by an individual.

The Spearman's rank correlation coefficient values suggest that although all distance measures are significantly related to one another (*p* < 0.01), the strength of this relationship becomes weaker as the spatial unit increases in size. This supports findings in the literature [9],[40],[41]. If individual level data is not available, we recommend that the smallest unit of aggregation be used. This is so that ecological fallacy is kept to a minimum and spatial variation can be modelled to a meaningful resolution.

This study has shown that in urban areas, if aggregate data is being used, the use of population weighted centroids produces smaller errors in measurements when combined with network measures of distance. However, if network distances are not available, Euclidean distance measures should be combined with geometric centroids. The combination of geometric centroids with Euclidean distances produces smaller distance errors than using population weighted centroids with Euclidean distances. The results of this study indicate that the use of geometric centroids with a Euclidean measure of distance produce more favourable results for rural areas. This is because the generalisation of the Euclidean geometric distances for LSOA and MSOA better represents the spatial variable of the distance travelled by the large population that is contained within these census units.

This study used an authoritative classification system [37] to stratify the data as urban or rural. It should, however, be acknowledged that the use of an alternate classification system could produce different results. In rural regions, where fewer people live and residential addresses are less densely clustered, or occur in pockets of clusters, geographical variation is more difficult to characterise in aggregate data than in urban areas. In the larger spatial units (MSOAs and LSOAs) in rural areas, spatial variation is smoothed to a greater extent. The differing stratification of morphologies may contribute to why previous studies have conflicting findings and to our knowledge the differences between urban and rural regions has not been reported before.

Defining rural and urban regions and recognising their differences are important for policy design and service planning [22]. It has been shown that characterising an area by its physical attributes at finer spatial resolution will allow for more detailed settlement types to be characterised [42], not just urban/rural regions. This may help planners, particularly in rural regions to better assess demand for a service. Rural areas tend to have poorer access to healthcare [43],[44] but by using small level aggregation units or, ideally, address data, accurate spatial distributions of populations can investigated which will give more accurate accessibility assessments [40].

To our knowledge, errors associated with network and Euclidean distances have not been quantified before. Quantifying the errors associated with commonly used distance methods will be a useful to public health practitioners and researchers who use these GIS methods to measure accessibility. Although there are more sophisticated methods available to calculate accessibility, network and Euclidean distances are a popular choice for public health practitioners and non-specialist GIS users. It is therefore important that users be aware of the error associated with their chosen method so that when analysing and the presenting results, the data is not assumed to be error free.

Further assessment of the distance methodologies examined the proportion of households that were assigned to the ‘correct’ GP. The correlation results show that based on distance from address to nearest GP, Euclidean distances are strongly correlated to network distances. However, at unit postcode level (r = 0.95 for Euclidean vs network distances), 12,000 more homes are sent to the wrong GP using the Euclidean method. This is an important consideration for cases where it matters which facility people are using and the assignment of individuals to services based on catchment areas. Depending on the methodology chosen there may be too few facilities in the most appropriate locations to meet demand. Conversely, over estimating the demand on a facility may lead to unnecessary resources being sent to a facility. In the context of facilities that treat chronic illnesses, the wrong assignment of households to the correct service centre could influence estimations on survival rates. A further consideration that must be taken in to account when using aggregate data is the ecological fallacy or “all or nothing” nature of assigning aggregate populations to the nearest facility. For example, at LSOA level 1500 people will all be routed to the same facility. For urban regions this had the most detrimental effect with up to 29,280 home being routed to the wrong facility at LSOA level. This is because there are more GP facilities in urban areas. Therefore within the aggregate unit there will be a greater variation in the GP that a population attend.

There are number of suggestions for further work and considerations to make: 1) Investigate facilities that are designed to serve larger populations, such as hospitals. It is likely that the correlation between Euclidean and network distances will be even weaker. This is because the number of natural and man-made barriers encountered on a longer journey, such as lakes and train lines will be greater. 2) We investigated accessibility to GPs which are expected to be within walking distance of under 4km [45]. Further work would be advised to consider topographic features of the local environment, such as elevation, en-route to facilities that are within walking distance. Topographic features may not be accurately captured when using the Euclidean method, and as such could be an important consideration that may reduce the correlation with network distances.

## 5. Conclusion

Although more sophisticated methods of accessibility are being and have been developed in research environments, the use of Euclidean and network distances remain a popular choice for modelling accessibility. The benefits and downfalls of these two distance methods have been well documented but the errors associated with the methodologies have not been quantified prior to this study. Further to the distance method introducing error in to accessibility modelling, aggregated data also produces errors. For future studies, the use of household level data should be encouraged; particularly in health studies. However, it should also be acknowledged that high resolution population data is often not available. No model is a perfect representation of the real world so it is important to acknowledge the error that is introduced by a methodology. In cases where aggregate data is being used, this study provides an aide-memoire that will allow practitioners and researchers to understand the implications of using particular data and methods.

## References

[1] Meyer SB, Luong TCN, Mamerow L, Ward PR (2013). Inequities in access to healthcare: analysis of national survey data across six Asia-Pacific countries. BMC Health Serv. Res.

[2] Ruiz-Casares M, Rousseau C, Derluyn I, Watters C, Crépeau F (2010). Right and access to healthcare for undocumented children: addressing the gap between international conventions and disparate implementations in North America and Europe. Soc. Sci.

[3] Stronks K, Ravelli A C, Reijneveld S A (2001). Immigrants in the Netherlands: equal access for equal needs?. J. Epidemiol. Community Health.

[4] Norredam M, Nielsen SS, Krasnik A (2010). Migrants' utilization of somatic healthcare services in Europe--a systematic review. Eur. J. Public Health.

[5] Meng Q, Xu L, Zhang Y (2012). Trends in access to health services and financial protection in China between 2003 and 2011: a cross-sectional study. Lancet.

[6] Gulliford M, Figueroa-Munoz J, Morgan M (2002). What does ‘access to health care’ mean?. J. Health Serv. Res Policy.

[7] Hu R, Dong S, Zhao Y, Hu H, Li Z (2013). Assessing potential spatial accessibility of health services in rural China: a case study of Donghai County. Int. J. Equity Health.

[8] (2010). Priorities for research on equity and health: Implications for global and national priority setting and the role of WHO to take the health equity research agenda forward. World Health Organisation. http://www.who.int/social_determinants/implementation/Thefinalreportnovember2010.pdf.

[9] Apparicio P, Abdelmajid M, Riva M, Shearmur R (2008). Comparing alternative approaches to measuring the geographical accessibility of urban health services: Distance types and aggregation-error issues. Int. J. Health Geogr.

[10] Luo W (2004). Using a GIS-based floating catchment method to assess areas with shortage of physicians. Health & Place.

[11] Lovett A, Haynes R, Sünnenberg G, Gale S (2002). Car travel time and accessibility by bus to general practitioner services: a study using patient registers and GIS. Soc. Sci. Med.

[12] Jordan H, Roderick P, Martin D, Barnett S (2004). Distance, rurality and the need for care: access to health services in South West England. Int. J. Health Geogr.

[13] Gatrell AC, Wood DJ (2012). Variation in geographic access to specialist inpatient hospices in England and Wales. Health & Place.

[14] Pearce J, Witten K, Bartie P (2006). Neighbourhoods and health: a GIS approach to measuring community resource accessibility. J. Epidemiol. Community Health.

[15] Gu W, Wang X, McGregor SE (2010). Optimization of preventive health care facility locations. Int. J. Health Geogr.

[16] Wang L, Roisman D (2011). Modeling Spatial Accessibility of Immigrants to Culturally Diverse Family Physicians. Prof. Geogr.

[17] Higgs G (2009). The role of GIS for health utilization studies: literature review. Heal. Serv. Outcomes Res. Methodol.

[18] Higgs G, Gould M (2001). Is there a role for GIS in the ‘new NHS’?. Health & Place.

[19] Luo W, Qi Y (2009). An enhanced two-step floating catchment area (E2SFCA) method for measuring spatial accessibility to primary care physicians. Health & Place.

[20] Langford M, Higgs G (2006). Measuring potential access to primary healthcare services: The influence of alternative spatial representations of population. Prof. Geogr.

[21] Using Geographic Information Systems for Health Research (2012). Application of Geogrpahic Information Systems. http://www.intechopen.com/books/application-of-geographic-information-systems/using-geographic-information-systems-for-health-research.

[22] Delamater PL, Messina JP, Shortridge AM, Grady SC (2012). Measuring geographic access to health care: raster and network-based methods.

[23] Boscoe FP, Henry K a, Zdeb MS (2012). A Nationwide Comparison of Driving Distance Versus Straight-Line Distance to Hospitals. Prof. Geogr.

[24] Goovaerts P (2009). Combining area-based and individual-level data in the geostatistical mapping of late-stage cancer incidence.

[25] Rodgers SE, Demmler JC, Dsilva R, Lyons RA (2012). Protecting health data privacy while using residence-based environment and demographic data. Health & Place.

[26] Omer I (2006). Evaluating accessibility using house-level data: A spatial equity perspective. Comput. Environ. Urban Syst.

[27] Ford D V, Jones KH, Verplancke J-P (2009). The SAIL Databank: building a national architecture for e-health research and evaluation. BMC Health Serv. Res.

[28] Luo L, McLafferty S, Wang F (2010). Analyzing spatial aggregation error in statistical models of late-stage cancer risk: a Monte Carlo simulation approach. Int. J. Health Geogr.

[29] Portnov B a, Dubnov J, Barchana M (2007). On ecological fallacy, assessment errors stemming from misguided variable selection, and the effect of aggregation on the outcome of epidemiological study. J. Expo. Sci. Environ. Epidemiol.

[30] Boone JE, Gordon-Larsen P, Stewart JD, Popkin BM (2008). Validation of a GIS facilities database: quantification and implications of error. Ann. Epidemiol.

[31] (2013). Population. http://www.swansea.gov.uk/population.

[32] Population Density (2011). Swansea - UK Census Data. http://www.ukcensusdata.com/swansea-w06000011/population-density-qs102ew#sthash.bRvQyPNs.YEwrd2zc.dpbs.

[33] NHS Wales Informatics Service - an Official NHS Wales website (2015). NHS Wales Informatics Service. http://www.wales.nhs.uk/sitesplus/956/home.

[34] (2014). Points of Interest. Ordnance Survey.

[35] (2014). AddressBase Premium. Ordnance Survey.

[36] (2014). Code-Point with polygons - locates every postcode unit in the UK with precision. Ordnance Survey.

[37] (2004). Rural and Urban Area Classification. ONS.

[38] (2014). OS MasterMap ITN Layer. Ordnance Survey.

[39] (2014). Census geography products for England and Wales. ONS.

[40] Goovaerts P (2013). Geostatistical Analysis of Health Data with Different Levels of Spatial Aggregation. Spat Spat. Epidemiol.

[41] Hewko J, Smoyer-Tomic KE, Hodgson MJ (2002). Measuring neighbourhood spatial accessibility to urban amenities: does aggregation error matter?. Environ. Plan. A.

[42] McLafferty SL (2003). GIS and health care. Annu. Rev. Public Health.

[43] Watt IS, Franks AJ, Sheldon T a (1994). Health and health care of rural populations in the UK: is it better or worse?. J. Epidemiol. Community Health.

[44] Campbell NC, Elliott a M, Sharp L (2000). Rural factors and survival from cancer: analysis of Scottish cancer registrations. Br. J. Cancer.

[45] Whitehouse C (1985). Effect of distance from surgery on consultation rates.

